# Integrating Physiological Data Artifacts Detection With Clinical Decision Support Systems: Observational Study

**DOI:** 10.2196/23495

**Published:** 2021-05-27

**Authors:** Shermeen Nizami, Carolyn McGregor AM, James Robert Green

**Affiliations:** 1 Systems and Computer Engineering Carleton University Ottawa, ON Canada; 2 University of Oshawa Institute of Technology Oshawa, ON Canada

**Keywords:** patient monitoring, clinical decision support, systems architecture, biomedical data analytics, alarm fatigue, physiological data artifacts

## Abstract

**Background:**

Clinical decision support systems (CDSS) have the potential to lower the patient mortality and morbidity rates. However, signal artifacts present in physiological data affect the reliability and accuracy of the CDSS. Moreover, patient monitors and other medical devices generate false alarms while processing physiological data, further leading to alarm fatigue because of increased noise levels, staff disruption, and staff desensitization in busy critical care environments. This adversely affects the quality of care at the patient bedside. Hence, artifact detection (AD) algorithms play a crucial role in assessing the quality of physiological data and mitigating the impact of these artifacts.

**Objective:**

The aim of this study is to evaluate a novel AD framework for integrating AD algorithms with CDSS. We designed the framework with features that support real-time implementation within critical care. In this study, we evaluated the framework and its features in a false alarm reduction study. We developed static framework component models, followed by dynamic framework compositions to formulate four CDSS. We evaluated these formulations using neonatal patient data and validated the six framework features: flexibility, reusability, signal quality indicator standardization, scalability, customizability, and real-time implementation support.

**Methods:**

We developed four exemplar static AD components with standardized requirements and provisions interfaces that facilitate the interoperability of framework components. These AD components were mixed and matched into four different AD compositions to mitigate the artifacts’ effects. We developed a novel static clinical event detection component that is integrated with each AD composition to formulate and evaluate a dynamic CDSS for peripheral oxygen saturation (SpO_2_) alarm generation. This study collected data from 11 patients with diverse pathologies in the neonatal intensive care unit. Collected data streams and corresponding alarms include pulse rate and SpO_2_ measured from a pulse oximeter (Masimo SET SmartPod) integrated with an Infinity Delta monitor and the heart rate derived from electrocardiography leads attached to a second Infinity Delta monitor.

**Results:**

A total of 119 SpO_2_ alarms were evaluated. The lowest achievable SpO_2_ false alarm rate was 39%, with a sensitivity of 80%. This demonstrates the framework’s utility in identifying the best possible dynamic composition to serve the clinical need for false SpO_2_ alarm reduction and subsequent alarm fatigue, given the limitations of a small sample size.

**Conclusions:**

The framework features, including reusability, signal quality indicator standardization, scalability, and customizability, allow the evaluation and comparison of novel CDSS formulations. The optimal solution for a CDSS can then be hard-coded and integrated within clinical workflows for real-time implementation. The flexibility to serve different clinical needs and standardized component interoperability of the framework supports the potential for a real-time clinical implementation of AD.

## Introduction

### Clinical Decision Support Systems

Clinical decision support systems (CDSS) are computerized health care analytic systems that have the functionality to integrate patient data for their analyses and detect clinically significant patient events. CDSS has the potential to lower patient mortality and morbidity rates when integrated into critical care workflows [1-5]. Clinical event detection (CED) algorithms that identify clinically significant events and early onset indicators of various pathophysiological diseases may be integrated into the CDSS to further exploit this potential [6-10]. Similarly, parameter derivation algorithms that extract clinically useful low-frequency parameters from high-frequency input data are also essential for clinical decision making [11-14]. However, the inherent presence of signal artifacts in physiological data impacts the reliability and accuracy of the analytical results produced by such algorithms [15]. Moreover, commercial physiologic patient monitors used in clinical settings are built using relatively simplistic proprietary algorithms for preprocessing artifacts [16-18]. This results in an unacceptably high rate of false alarms generated by these patient monitors [19]. Such alarms, termed as nuisance or false alarms, result in increased noise levels, staff disruption, and staff desensitization in busy critical care environments [20-22]. False alarms need to be typically silenced or overridden by staff, which leads to alarm fatigue, causing an even bigger hazard of missed alarms and compromising the quality of care at the patient bedside [21,23,24]. The Emergency Care Research Institute, a Pennsylvania-based patient safety organization, issued an annual report of the top 10 health technology hazards. Leading up to and including 2019, the Emergency Care Research Institute has reported medical device alarms to be among the top 10 hazards. The literature has reported false alarm rates (FAR) greater than 70% [25]. The integrity and quality of data are crucial to the success of any analytic system. Therefore, it is important to design and implement CDSS for assessing the quality of data and issue relevant alarms with a high specificity and low FARs. A recent study suggested behavioral methods to reduce false alarms and alarm fatigue in the neonatal intensive care unit (NICU) [26]. The study was conducted in an NICU in a low-income country (India) [26], whereas our study was conducted in a high-income country (Canada) where the recommended behavioral changes have already been implemented [27].

### Artifact Detection

Research groups have published several artifact detection (AD) algorithms to assess the quality of physiologic data and minimize the impact of artifacts before analyzing these data for CED. However, a methodological literature review by the authors conveys common limitations in the application of a vast majority of AD algorithms [28]. In this review, we synthesized more than 80 state-of-the-art AD algorithms and discovered the following six shortcomings: most AD algorithms (1) are designed for one specific type of critical care patient population, (2) are validated on data harvested from a single monitor model, (3) generate signal quality indicators (SQIs) that are not yet standardized for useful integration in clinical workflows, (4) operate either in standalone mode or are tightly coupled with other CDSS applications, (5) are rarely evaluated in the real time, and (6) are not implemented in clinical practice [28]. A more recent review on the initiatives to manage and improve alarm systems taken by means of human, organizational, and technical factors for an improved quality of health care also supports our findings [20]. The review reveals gaps between alarm-related standards and how those standards are translated into practice, especially in a clinical environment that uses multiple alarming medical devices from different manufacturers [20]. This suggests standardization across devices from the same and different manufacturers and the use of machine learning to improve the alarm safety [20].

### AD Framework

To address the six shortcomings (1)-(6) that are listed above, we designed and developed a novel, multivariate, component-based, standardized AD framework [29]. For the reader’s convenience, the *Methods* section provides the background on framework development, including the design of its components and interfaces by developing a common reference model (CRM). The objective is to facilitate the integration of AD and CED algorithms within the CDSS in a standardized manner. To achieve this, we leveraged six framework features *f1 to f6*, which are listed in then Methods section. We designed the AD framework as a test bed to formulate and evaluate multiple combinations of independently developed AD and CED components. Once a combination of AD and CED is affirmed to satisfy clinical needs through offline testing, then that combination can be evaluated in a real-time environment using the middleware technology. In this way, the transition to real-time clinical implementation and validation can be facilitated by using this framework.

For the reader’s convenience, this section summarizes the development of the AD framework, as in a previous study [29]. This section comprises the development of the components and interfaces that provide the framework’s end-to-end functionality, a CRM for the standardized communication between components across their interfaces and the framework’s features.

#### Components and Interfaces

A framework comprises components that interact with each other and with the system through one or more interfaces to realize the system goals. An *interface* is defined as a means of communicating with or accessing a component [30]. Clearly defined uniform interfaces enable components to make their own functional requirements explicit as well as to enable specifications of other collaborating components. Interfaces stipulate prerequisites, provisions, and constraints of component operations. A component can have one or more interfaces, selectively instantiated at the runtime depending on the component’s role in a particular composition. As described in a previous study [31], an interface can be categorized as (1) requirement, (2) provision, and (3) configuration. Each component has its own operational requirements, specified by its requirement interface, which defines what the system or other components in the system must provide for the component to function [30]. The provision interface makes explicit what a component can provide either to another component or as a contribution to the system output. The configuration interface incorporates a user-defined functionality, further allowing the user to define the runtime parameters for a particular application. A configuration interface can be part of the user interface designed for a clinician to interact with the system settings.

The AD framework comprises the following components: (1) patient data acquisition (PDA), (2) AD, and (3) CED. Each component is composed of low-level code and the following three interfaces: (1) requirement, (2) provision, and (3) configuration. Framework components can interface as either standalone algorithms or in cascade with the same or different types of components.

#### Common Reference Model

**Figure 1:**
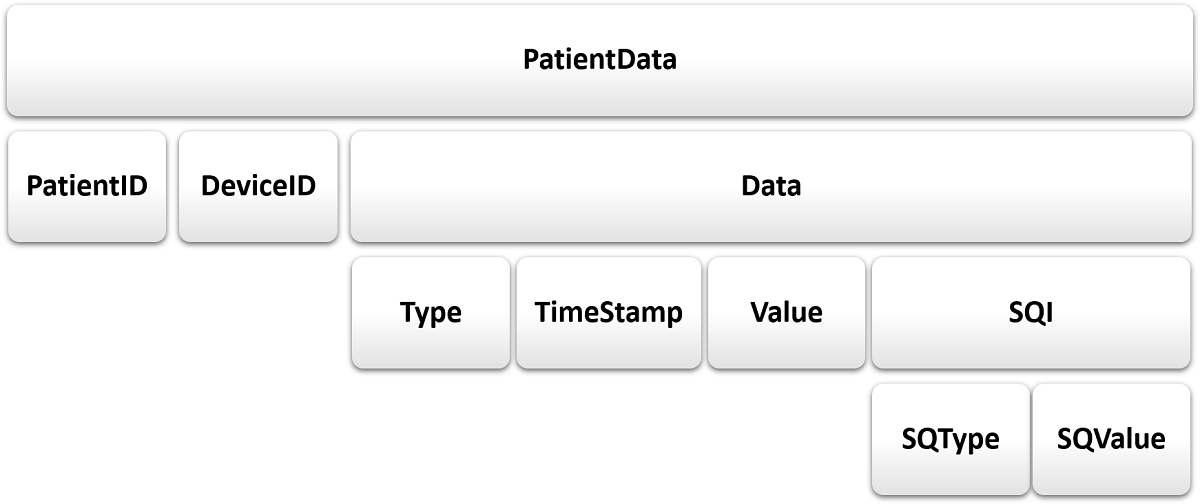
Common reference model schema consisting of the patient data metadata used by each component’s requirement and provision interfaces at input and output. SQ: signal quality; SQI: signal quality indicator.

The standardization of interfaces is key for achieving the system goals. This involves defining unambiguous formalisms with semantics that are commonly understood by all components within the framework. A novel CRM was developed to standardize the definitions for these interfaces to facilitate component interoperability within the AD framework [29]. Multiple medical ontologies are in existence to address the measurement of medical parameters such as LOINC (Logical Observation Identifiers Names and Codes), which is a database and universal standard for identifying medical laboratory observations; Systematized Nomenclature of Human Medicine (SNOMED), which is a multiaxial nomenclature for indexing medical records; and the Fast Healthcare Interoperability Resources which is an interoperability standard created by the standards development organization Health Level 7 to enable health data, including clinical and administrative data, to be quickly and efficiently exchanged across medical devices. The CRM interfaces designed as a part of our framework are easily customizable to match any of these standards. CRM comprises metadata that are intended to establish a common understanding of the meaning or semantics of the data exchanged between component interfaces. This allows all framework components to communicate, regardless of their underlying low-level logic. For example, CRM facilitates interfacing a variety of AD algorithms for different types, frequencies, and quality of physiologic data that are commonly processed by CDSS. In particular, the standardization of SQIs is a novel contribution to the development of CRM. The CRM metadata comprise the following layered schema: PatientData (*PatientID*, *DeviceID*, *Data* (*Type*, *TimeStamp*, *Value*, *SQI (SQType* and *SQValue*)))*.* PatientData represents the patient data exchanged between the components. Its schema consists of three properties, as shown in Figure 1 (*PatientID, DeviceID*, and *Data*). *PatientID* identifies the patients with whom the data are associated. It can be any type of patient identifier, such as the patient’s admission reference number. *DeviceID* represents the hospital or original equipment manufacturer (OEM) identifier for the patient monitor or other devices from which the data are being acquired. The more complex *Data* property has the following four attributes: *Type, TimeStamp, Value*, and *SQI*. *Type* is a string variable from a controlled schema, naming the physiological data stream. *TimeStamp* is the time at which each datum is logged. A component may have specific data exchange and processing rates, which require data at specific frequencies. Therefore, *TimeStamp* can be used to (1) derive the frequency of data, (2) align multiple data streams for fusion, and (3) annotate events in real time. *Value* contains the numeric or string value of each datum. An *SQI* may also be associated with each datum. This measure of signal quality is provided by the monitor (via a PDA component) or derived by one or more AD algorithms. The *SQI* for each datum is further described by two attributes: *SQType* and *SQValue*. *SQType* is a string variable from a controlled schema, for example, “binary,” “rank,” “categorical,” or “null.” New strings can be introduced in this set in the future. “Null” implies there is no SQI available for that particular data type. *SQValue* depends on *SQType*. For example, if *SQType* is “binary,” then *SQValue* belongs to a set of 0 or 1. This schema is extensible when needed for newer CDSS formulations. Our preliminary research demonstrates the instantiation of CRM using XML [32].

At runtime, the PDA component inputs patient data and conforms them to the CRM, which are then consumed by the AD and CED components that comprise the CDSS. AD and CED algorithms, published in the past or future, whether standalone or tightly coupled, may be used in CDSS formulations with modifications as needed. The framework is a unique test bed with features of reusability and scalability. These features allow for the creation of new AD configurations by mixing and matching independently developed or decoupled AD components and integrating those components with CED components to serve varying clinical needs. The AD configuration most suited to a clinical need can then be hard-coded and integrated into the clinical workflow for real-time implementation. For example, some recently developed AD algorithms leverage sensor fusion for motion artifact removal while deriving the heart rate (HR) [33-37]. The implementation of these AD and CED algorithms within the framework simply requires modifying their interfaces to comply with the CRM. This would allow for these algorithms to be tested, compared, or combined with extant or newer algorithms to advance research in the field of signal quality and physiologic monitoring.

#### Framework Features

To address the six shortcomings (1)-(6) identified in state-of-the-art AD algorithms in a previous study [28], we developed an AD framework with the following six features *f1* to *f6* The framework design supports: (*f1*) flexibility to serve the needs of patient populations from different types of critical care units through generalization and customizability, (*f2*) reusability across multiple types of physiological data harvested by different OEM monitors, (*f3*) standardized definitions of SQI that promote interoperability and comparison between independently developed components, (*f4*) reusability and scalability by mixing and matching several AD and CED components in various combinations, (*f5*) customizability to evaluate and compare the performance of multiple combinations of independently developed components on offline and potentially real-time patient data when integrated with clinical workflow, and (*f6*) standardized component interfaces that can potentially support real-time clinical implementation of AD. This study validates the six framework features *f1* to *f6*.

### Research Contribution

The main contribution of this paper is the dynamic evaluation of the AD framework as a test bed, given the clinical context of false alarm reduction in medical devices. In this study, we first developed a catalog of several exemplar AD components and a single CED component. The interfaces of all these components comply with the CRM, such that they can be integrated within the AD framework. Given the motivation for false alarm reduction, we designed a novel CED component that can generate peripheral oxygen saturation (SpO_2_) alarms. We then created four unique CDSS configurations by mixing and matching different AD components from the catalog with the same SpO_2_ alarm generation CED component. The *Methods* section describes the research methodology, including the development of the framework component catalog and the four CDSS formulations used for evaluating the framework and its features. This section demonstrates how the framework leverages existing AD algorithms by incorporating them with the SpO_2_ alarm–generating CED component. The four configurations are designed and evaluated based on the results and recommendations in the state-of-the-art research linked to the reduction of false alarms generated by OEM monitors. Although CRM has been developed after an extensive review of the literature that summarizes the requirements, provisions, and configurations for many existing AD algorithms, it is expected that the CRM will continue to evolve because a wide variety of new AD and CED algorithms with differing data needs are implemented as components within this framework. For example, a new OEM alarm management system, Philips Care Event, was evaluated along with the optimization of the clinical workflow in the NICU [25]. The OEM system delay time for saturation-related alarms was increased from 10 to 20 seconds, and the averaging time was decreased from 10 to 4 seconds without changing the standard alarm settings. This strategy led to a reduction in the number of SpO_2_≤80% alarms and an increase in nurses’ response to alarms [25]. This is an exemplar state-of-the-art CED strategy that can be easily accommodated and evaluated in combination with various AD techniques using our framework to further reduce false alarms and subsequent alarm fatigue. In this way, the framework can facilitate the discovery of optimal CDSS formulations through the mixing and matching of new AD and CED components supported by an evolving CRM.

*Methods* section describes the framework evaluation methodology comprising the data collection method and performance evaluation metrics of sensitivity (Sn) and FAR. For framework validation, we used real patient data collected from 11 neonates during a clinical study at the NICU of the Children’s Hospital of Eastern Ontario (CHEO), Ottawa, Ontario, Canada. Harvested data streams include HR, pulse rate (PR), SpO_2_, and their corresponding alarms from physiologic patient monitors. Several conditions, such as hypothermia (peripheral vasoconstriction), edema (increased thickness and, therefore, diffusion distance for oxygen), increased skin pigmentation, and shock, are known to decrease the clinical reliability of SpO_2_. None of the patients in this study had any such condition.

*Results* section provides the performance evaluation results in terms of Sn and FAR of the SpO_2_ alarms generated by each of the four CDSS formulations. Once a CDSS formulation is affirmed to satisfy clinical needs through offline testing by applying this methodology, the optimal combination can be evaluated in a real-time environment using the middleware technology. This will facilitate the real-time implementation of the optimal CDSS formulation through hard-coded integration within clinical workflows.

It should be noted that all four CDSS formulations deploy the same CED component for SpO_2_ alarm generation. Hence, the sensitivity of the CED component to the error profiles and the impact of errors remain controlled or constant across all experiments. Therefore, the reported Sn and FAR values reflect the performance of the four different AD configurations, regardless of the performance of the CED component. In other words, the framework evaluation reported here remains independent of the performance of the CED component. This validates the use of the framework as a test bed to discover the optimal combination of AD components with a CED component that is designed for a specific clinical problem. In the future, the framework can be similarly deployed with another CED component for different clinical problems.

*Discussion* section discusses the research contributions and provides a detailed discussion on the validation of the six framework features (*f1*) to (*f6*). Section 7 concludes the paper and suggests directions for future work.

## Methods

### Overview

According to Larsen [30], beyond designing and building a component-based framework, its evaluation requires static models that illustrate component structures as well as dynamic models that illustrate component collaboration. This paper first develops a catalog of static PDA, AD, and CED components. Subsequently, four dynamic compositions of these components were formulated and evaluated using real patient data. Each of the AD components processes physiological data streams in the form of numeric or string values, and the CED component generates alarms on the SpO_2_ data stream. The requirements and provision interfaces of all components comply with the CRM, such that they can be integrated within the AD framework. Each configuration is integrated with PDA and CED components to formulate a CDSS that generates SpO_2_ alarms at its output.

The following subsections expand upon this research methodology: *Components Catalog* develops a catalog of framework components; *CDSS Formulations* mixes and matches these components to build and evaluate four different CDSS formulations; and the *Evaluation* subsection uses real patient data to evaluate the performance of each CDSS formulation, thereby validating the use of the framework as a test bed; and determining the optimal CDSS formulation for SpO_2_ alarm generation. Once a combination is affirmed to satisfy clinical needs through offline testing by applying this method, the optimal combination can be evaluated in a real-time environment using the middleware technology. This will facilitate the real-time implementation of the optimal CDSS formulation through hard-coded integration within clinical workflows.

### Components Catalog

In this subsection, we develop a catalog of framework components comprising an original PDA component, four AD components, and one novel CED component. The catalog represents a model instantiation of the framework comprising the original PDA and CED components designed in collaboration with our clinical partners. The catalog is not meant to represent an exhaustive or particularly novel set of AD components; rather, it tailors the interfaces of existing AD algorithms to comply with the CRM.

#### PDA Component

**Figure 2:**
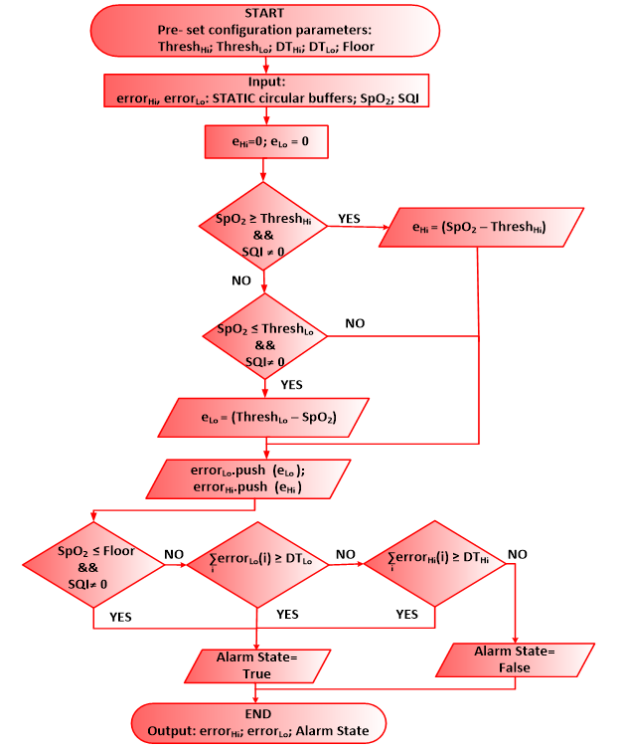
Low-level component code for clinical event detection and generation of peripheral oxygen saturation alarms. SpO2: peripheral oxygen saturation; SQI: signal quality indicator.

As defined in our earlier research, the PDA component inputs patient data from sources that include, but are not limited to, OEM patient monitors, clinical data entry, lab results, physician’s order, and patient demographics from electronic health records [29]. In this research, the PDA inputs the physiological data and alarm streams from the OEM monitors and translates these data to the schema defined by the CRM. It then feeds these data to one or more AD components, as shown in the CDSS flowcharts in Figure 2. In these workflows, the hardware and software requirements are factored in the PDA component. The hardware comprises the Digi International Edgeport4 (Digi International), which consists of the Eltima Port Monitor Professional Edition Software v4.x (Eltima Software) for data logging with additional customized software written in JAVA to conform the OEM-generated data streams to the CRM. Specifically, the *Data.Type* (SpO_2_, HR, PR, and alarm status) and corresponding *Data.Values* were extracted from each interleaved OEM data packet. Each packet was produced by the monitor at 0.5 Hz. The low-level code of the PDA component interpolated and synchronized the data streams at 1 Hz. As the OEM monitors fail to provide an explicit SQI stream for any of the data types, a default SQI stream with a *SQType*=“binary” and *SQValue*=1 is generated by the PDA component for each data type using MATLAB (MathWorks).

#### AD Components

We surveyed a wide variety of techniques used by AD algorithms to detect, mitigate, and suppress physiological artifacts that are found in clinical settings [28]. To demonstrate the framework composition, we developed four AD components exemplifying the following diverse AD functionalities: (1) data and SQI deinterlacing, (2) SQI fusion, (3) data fusion, and (4) data smoothing. Although each exemplar component differs in its low-level code, all components conform to the CRM. The low-level code and configuration interfaces for each functional group of the components are described as follows.

#### ADDIL DeInterlace Component

Some monitors produce a single output stream, which is, in fact, interlaced with the data and SQI. The AD_DIL_ component is designed to deinterlace (*DIL*) these two information streams by allowing the user to define a set of symbols (*artSyms*) to be associated with the corresponding SQI values. Typically, *artSyms* is a list of artifact indicators specified by the manufacturer, which could be either numeric or string values that replace the value of the datum. For example, for Infinity monitors (Dräger Medical Systems), the set of *artSyms* would include {NaN,^^,5}, where Not-a-number (NaN) is substituted for any missing datum, ^^ is an artifact indicator, and 5 is an alarm state (ie, part of the alarms stream) indicating a lead disconnection. Therefore, a data segment interlaced with artifacts is logged with the corresponding *artSyms* value. In a different example, Philips Intellivue MP70 monitors (Philips) generate a value of “2” in the alarms data stream in case of leads disconnection. However, with the alarm data stream connected to the input of the AD_DIL_ component, the value “2” can be identified by the component as an *artSyms*. In such a way, the component can deinterlace the alarms stream and generate a corresponding binary SQI stream, where the value “2” would be replaced by a 0. The low-level code for AD_DIL_ is given by equation (1).

if (Data.Value(i) artSyms); SQI_out_(i)=SQI_Match_(Data.Value(i)); end **(1)**

The configuration interface of the AD_DIL_ component specifies the Data.Type to be examined, *artSyms*, and the corresponding set of *SQValue* (SQI_Match_). This AD component produces a “rank” *SQType*, with “binary” being a special case of “rank,” where SQI_Match_=0. Multiple instances of this component were cascaded in the AD framework in this validation study.

#### ADFuseSQI Fuse SQI Component

The AD_FuseSQI_ component accepts more than one data stream at its requirements interface, along with the respective SQI of each stream. This component combines *N* incoming SQI inputs to generate a single fused SQI (*FuseSQI*). The fused SQI value is equal to the operator, that is, the minimum, maximum, or average SQI value from all the input SQI data at any given instant. This requires all the input *SQTypes* to be the same. The low-level code for AD_FuseSQI_ is shown in equation (2).

SQI_out_(i)=operator (SQI_1_, SQI_2_,..., SQI_N_) **(2)**

The configuration interface of the AD_FuseSQI_ component defines *N*, the required input *SQType* (same as output), and the operator (min, max, and avg) to be applied to all input SQI values. In addition, the configuration interface can specify which data types to forward at the provision interface, as only a subset of the input streams may be required beyond this component. Equation (2) is a relatively simple depiction of data fusion. Data can be fused at different levels of abstraction, requiring a more complex combination of operators and weighting [38].

#### ADDiff Differential Component

The AD_Diff_ component calculates an absolute differential error function between two input data streams, Data_1_ and Data_2_. This error value was then compared with a configured threshold. The input “binary” SQI streams are examined such that if either stream has a poor signal quality, then the output *SQValue*=0. This component can be used in the case where two independent measurements of the same physiological parameter are provided; then, this component will derive an SQI by exploiting data fusion. The configuration interface specifies the *output SQType* to be produced; the *Data.Type* of Data_1_ and Data_2_; the number of SQI thresholds, n_Thresh_, to be applied to the difference; the ordered set of thresholds (*SQThresh_j_;j=1:*n_Thresh_); and the set of n_Thresh_+1 *SQValues* (SQI_j_) corresponding to each threshold with the additional *SQValue* for the default case (*SQI_default_)*. The configuration interface can specify which data types to forward at the provision interface. The low-level code for this component is illustrated in equation (3), as follows:

SQI_out_ (i)=*SQI_default_*;

if (Data_1_.SQI.SQValue(i)==0) || (Data_2_.SQI.SQValue(i)==0);

return; diff=|Data_1_.Value(i)−Data_2_.Value(i)|;

for j=1: n_Thresh_

{if (diff ≤ *SQThresh*_j_){SQI_out_(i)=SQI_j_;break;}}end **(3)**

The AD_Diff_ component can derive a “rank” *SQType* stream from HR and PR streams by configuring the component to have *output SQType* set to “rank”; the *Data.Type* of Data_1_=HR and Data_2_=PR; n_Thresh_=3; *SQThresh*={6,12,18}; and SQI_j_={3,2,1,0}, where the *SQI_default_*=0. This configuration of the AD_Diff_ component was used in the validation study.

For example, consider the work on wearable devices and systems published by He et al [39]. Their study synchronously collected the data of ballistocardiogram, electrocardiography (ECG), and photoplethysmography. Their study suggests checking if all three physiological signals measure the same values for HR so that this information can be used to ensure that the acquired data are not corrupted. However, their study did not demonstrate whether and how it checks for data quality. Such a system would benefit from using the AD_Diff_ component.

#### ADMedFilt Median Filter Component

The AD_MedFilt_ component implements a median filter (*MedFilt*). It is used for smoothing a stream of data to mediate abrupt transient artifacts. The configuration interface defines the size of the sliding window *Med_WW_* for use while computing the median value. Its requirement interface inputs a single data type and its corresponding SQI stream. Each datum in the output data stream was equal to the median of the past *Med_WW_* input data samples. Only a subset of these *Med_WW_* may actually be used in computing the median because the AD_MedFilt_ component only includes the data within the sliding window for which the input SQI is acceptable. The SQI stream passed through this component and remained unchanged. By comparing the filtered and unfiltered data using an AD_Diff_ component, one can compute an SQI proportional to the degree of smoothing applied to each point. The AD_MedFilt_ component was used in CDSS formulations in this study.

#### CED Component

In this subsection, we develop a novel CED component that generates SpO_2_ alarms. By discussing and reaching consensus with our clinical collaborators at CHEO, we translated clinical rules into low-level logic to create a CED component with a requirements interface that conforms to the CRM. Alarm generation studies suggest these two approaches to reduce the FAR: (1) modifying or adjusting the alarm thresholds and (2) introducing alarm annunciation delays, that is, a delay between when an alarm threshold is crossed and when the alert is sounded or displayed [25,40-43]. These studies test alarm annunciation delays anywhere from 5 to 120 seconds for a variety of physiological data types. However, none of these studies quantify the trade-off between Sn and FAR resulting from their suggested alarm generation algorithms. In our study, the CED component incorporates both approaches described above to reduce FAR. Its low-level code allows for adjusting the alarm thresholds by reduction in the lower SpO_2_ alarm threshold and increment in the upper SpO_2_ alarm threshold. During evaluation, both limits were adjusted by 3%, which corresponds to the manufacturer-specified margin of error in the accuracy of the pulse oximeter reading. Therefore, the low alarm threshold, *Thresh_Lo_*, is breached if the SpO_2_ value is lower than the alarm threshold of the OEM monitor by at least 3%, and the upper alarm threshold *Thresh_Hi_* is breached if the SpO_2_ value is higher than the alarm threshold of the OEM monitor by at least 3%. Incorporating the second approach, the low-level code of the CED allows for tuning the alarm annunciation delays (CED_DT_) between 5 and 60 seconds.

Figure 2 shows a flowchart of the low-level source code of the CED component. In this case, the user is an expert who composes the CDSS in collaboration with the clinician. The user can set tunable parameters at the configuration interface, including values for *Thresh_Lo_*, *Thresh_Hi_*, DT_LO_, DT_HI_, and Floor. Floor is an absolute minimum SpO_2_ value determined by clinicians, typically in the range of 50%-75%. We set a Floor value below because SpO_2_ sensors are unable to calibrate at such low values; hence, the true state of the patient can no longer be determined, and the CED must alarm to alert the clinician to come and check the patient. The CED continuously compares the SpO_2_ value with the lower and upper limits, *Thresh_Lo_* and *Thresh_Hi_*, respectively. A history of threshold breaches gets stored in circular buffers, error_Lo_ and error_Hi_. These breaches are summed over a sliding window such that the total error is a function of both the magnitude and duration of the threshold breaches. The integrated error is continuously compared with the tunable lower and upper decision thresholds, DT_LO_ and DT_HI._ These decision thresholds are set proportional to the CED_DT_ value, which is set at the configuration interface of the CED component. Specifically, DT_LO_ is set equal to CED_DT_, and DT_HI_ is set to twice the CED_DT_ because high SpO_2_ alarms are not clinically deemed to be as dangerous as low SpO_2_ alarms. Therefore, the CED waits twice as long to generate a high SpO_2_ alarm as compared with a low SpO_2_ alarm. The decision to generate an alarm is based on three conditions, as shown in Figure 2. The CED generates an alarm if the incoming SpO_2_ value is less than or equal to the set value of Floor and the incoming SQI is not zero, or if the integrated errors, namely error_Lo_ or error_Hi_, exceed DT_LO_ or DT_HI_, respectively. Here, we configured parameters suitable for the neonatal population. Users may tune the parameters specific to other patient populations.

### CDSS Formulations

This section describes the dynamic framework compositions of the four CDSS formulations. MATLAB was used for the dynamic framework modeling. Table 1 lists the requirements, provisions, and configuration interfaces for each AD component deployed in the four CDSS formulations.

**Table 1:** Artifact detection component interfaces used in clinical decision support systems formulations.

AD^a^ component			Interface				
			Requirements		Provisions		Configuration
**CDSS^b^ #1 and CDSS #2**							
	AD_Dil_	[SpO_2_^c^ Alarms, SQI^d^]		[SpO_2_Alarms, SQI]		*artSyms*^e^{NaN^f^,^^,5}	
	AD_Dil_	[SpO_2_, SQI]		[SpO_2_, SQI]		*artSyms*{NaN,^^,5}	
	AD_FuseSQI_	[SpO_2_ Alarms, SQI]; [SpO_2_, SQI]		[SpO_2_, SQI]		N=2; operator (min); *SQType*^g^=*“*binary”	
**CDSS #2 (** **additional component** **)**							
	AD_MedFilt_	[SpO_2_, SQI]		[SpO_2Med_, SQI]		*Med*_WW_^h^ *={5,10,20,25,30,35,60}*	
**CDSS** **#** **3** **and** **CDSS** **#4**							
	AD_Dil_	[HR^i^, SQI]		[HR, SQI]		*artSyms*{NaN,^^,5}	
	AD_Dil_	[PR^j^, SQI]		[PR, SQI]		*artSyms*{NaN,^^,5}	
	AD_Dil_	[SpO_2_, SQI]		[SpO_2_, SQI]		*artSyms*{NaN,^^,5}	
	AD_Dil_	[SpO_2_Alarms, SQI]		[SpO_2_ Alarms, SQI]		*artSyms*{NaN,^^,5}	
	AD_FuseSQI_	[SpO_2_Alarms, SQI]; [SpO_2,_ SQI]		[SpO_2,_ SQI]		*N*=2;operator(min); *SQType*=*“*binary”	
	AD_Diff_	[HR, SQI]; [PR, SQI]		[PR, SQI]		*Data1.Type=“HR”;Data2.Type=“PR”;SQType*=*“*binary”; *SQThresh*={6,12,18};*SQI*_default_*=0*	
	AD_FuseSQI_	[SpO_2,_ SQI]; [PR, SQI]		[SpO_2,_ SQI];		N=2; operator(min); *SQType*=*“*binary”	
**CDSS** **#** **4** **(** **additional component)**							
	AD_MedFilt_	[SpO_2,_ SQI]		[SpO_2Med_, SQI]		*Med*_WW_ *={5,10,20,25,30,35,60}*	

^a^AD: artifact detection.

^b^CDSS: clinical decision support systems.

^c^SpO_2_: peripheral oxygen saturation.

^d^SQI: signal quality indicator.

^e^*artSyms*: a list of artifact indicators with corresponding values of SQI specified by the manufacturer.

^f^NaN: Not-a-number.

^g^SQType: a string variable from a controlled schema with corresponding types of SQI.

^h^Med_WW_: size of the sliding window of the median filter.

^i^HR: heart rate.

^j^PR: pulse rate.

#### CDSS #1

**Figure 3:**
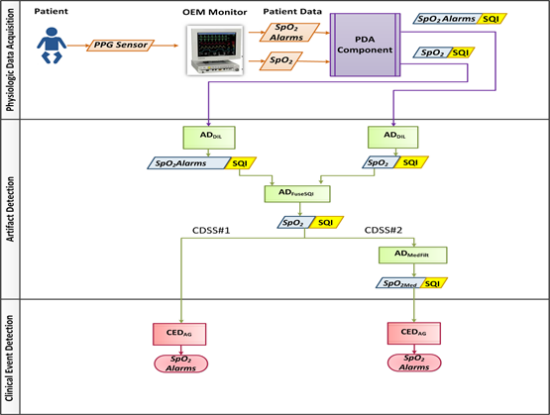
Flowchart showing the patient data acquisition, artifact detection, and clinical event detection components in clinical decision support systems CDSS # 1 and 2 formulations. CDSS: clinical decision support systems; ECG: electrocardiography; HR: heart rate; PDA: patient data acquisition; PPG: photoplethysmography; PR: pulse rate; SpO2: peripheral oxygen saturation; SQI: signal quality indicator.

CDSS #1 constitutes the simplest of the four compositions designed for this study. A flowchart for CDSS #1 is shown in Figure 3. This flowchart has three functional horizontal swim lanes, depicting the PDA, AD, and CED components of the integrated CDSS. Each data stream is represented by a tuple with both data and SQI information. The input data stream is sourced only by the SpO_2_ sensor comprising two data types, namely, SpO_2_ and SpO_2_ alarm status (*SpO_2_Alarm*). The low-level logic of the PDA component maps the incoming values to its respective data type (*SpO_2_ or SpO_2_Alarm*) and assigns a default *SQValue* of 1 to each datum of each *Data.Type* because an SQI value is not provided by the OEM monitor in this case.

The AD composition pipeline in CDSS #1 consists of two ADDILs and one AD_FuseSQI_ component. The AD_DIL_ component deinterlaces the OEM-generated artifacts, whereas the AD_FuseSQI_ component combines the SQI streams from the two AD_DIL_ components. The PDA provides *SpO_2_* and its associated *SQI* stream to one instantiation of AD_DIL_ while providing *SpO_2_Alarm* and its *SQI* stream to the second instantiation of AD_DIL_. The low-level code of the AD_DIL_ component deinterlaces the OEM-specified artifact values. Here, the user-set configuration interface includes *artSyms=*{NaN,^^,5} and SQI_Match_=0. The “NaN” string implies missing data, and the “^^” symbol represents OEM-specified artifact values in the *SpO_2_* stream, whereas “5” is interlaced within the *SpO_2_Alarm* to imply that the SpO_2_ sensor is off. Hence, the use of the two AD_DIL_ components would provide the original data streams of *Data.Type* *SpO_2_* and *SpO_2_Alarm*, along with their respective *SQI* streams, with *SQValue*=0 wherever the *Data.Value* is equal to any one of the *artSyms*. These 2 data streams and their associated *SQI* streams are then input to the requirements interface of an AD_FuseSQI_ component. The low-level code of the AD_FuseSQI_ component fuses two or more incoming *SQI* inputs to generate a single fused *SQI* value. In this formulation, the operator is set to *min*; hence, it provides an output *SQValue* that is the minimum of the 2 input *SQValue* for which *SQType*=“binary*.*” As shown in Figure 3, this output SQI stream is associated with the original SpO_2_ stream that is required by the CED component.

#### CDSS #2

CDSS #2 extends the CDSS #1 formulation by adding an AD_MedFilt_ component to process the *SpO_2_* data stream through a median filter for reducing transient artifacts. This extension is labeled CDSS #2 in Figure 3. The low-level code of the AD_MedFilt_ configuration interface comprises a tunable parameter *Med_WW_*_=_{*5*,*10*,*20*,*25*,*30*,*35*,*60*}, and the component produces a median filtered *SpO_2Med_* data stream and its associated SQI stream, which are then passed to the requirements interface of the CED component.

#### CDSS #3

**Figure 4:**
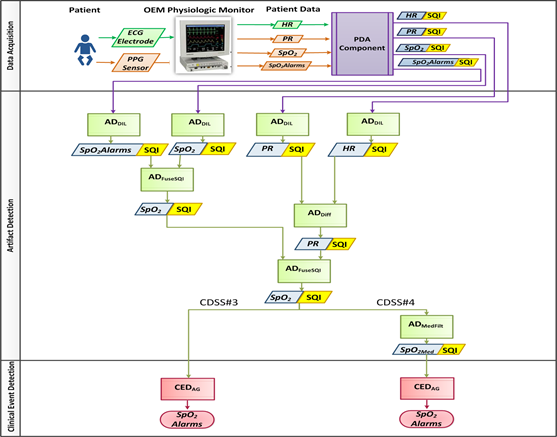
Flowchart showing the patient data acquisition, artifact detection, and clinical event detection components in clinical decision support systems CDSS # 3and 4 formulations. CDSS: clinical decision support systems; ECG: electrocardiography; HR: heart rate; PDA: patient data acquisition; PPG: photoplethysmography; PR: pulse rate; SpO2: peripheral oxygen saturation; SQI: signal quality indicator.

CDSS #3 leverages data fusion to derive an estimate of the signal quality for *SpO*_2_. Here, an AD_Diff_ component computes the difference between the PR and HR. Physiologically, PR and HR are equal, representing the mechanical and electrical pumping rates of the heart, respectively. Therefore, any difference between PR and HR serves as a proxy for signal quality measurements. In this study, HR is considered as the gold standard. Therefore, a large difference between the instantaneous PR and HR values indicates that the PR has deviated and is not reliable. In this case, a low SQI is assigned to both PR and SpO_2_ because both are sourced from the same sensor. Figure 4 shows the PDA, AD, and CED components in the flowchart for CDSS #3. The low-level code of the AD_Diff_ component computes the difference between the instantaneous HR and PR values. By comparing that difference to a threshold, a “binary” *SQType* is generated, which is then passed to the requirements interface of the CED component. The configuration interface was set with a single threshold to produce a “binary” *SQType*. The SQI threshold (*SQThresh*) is varied in the range {6,12,18} to examine its effect, and the results are reported separately for each.

#### CDSS #4

CDSS #4 builds on the composition of CDSS #3, as depicted in Figure 4. Here, an AD_MedFilt_ component is added such that the *SpO_2_* data stream can be median filtered to produce SpO_2Med_ data and SQI streams, which are then fed to the requirements interface of the CED component. The tuned values of *Med_WW_* include {5,10,20,25,30,35,60}.

### Evaluation

#### Clinical Data Collection

Data were collected during a clinical study conducted in the CHEO NICU. The study was approved by the hospital’s Research Ethics Board. In total, 11 neonatal patients with diverse pathologies were enrolled in this study. The following time-stamped data streams and corresponding alarms were collected simultaneously from each infant at a frequency of one reading every 2 seconds (0.5 Hz): PR and SpO_2_ from a pulse oximeter (Masimo SET SmartPod Model # MS16356, Masimo Corp) integrated with an Infinity Delta monitor (Dräger Medical Systems) as well as HR derived from ECG leads attached to a second Infinity Delta monitor. HR and PR are parameters that estimate the rate at which the heart beats per min (bpm). Although HR and PR are acquired independent of each other, they essentially represent the exact same functionality of the heart, albeit in electrical and mechanical contexts, respectively. HR is acquired through ECG leads, which are electrical sensors, and PR is acquired using optical sensors attached to the pulse oximeter. Moreover, the pulse oximeter derives SpO_2_ using the same optical sensor data. This implies that the quality of the PR data stream reflects the quality of the SpO_2_ data stream. Therefore, to evaluate the framework as a CDSS that generates SpO_2_ alarms, we selected the HR as the gold standard for comparison with the quality of the PR data stream. The reason for selecting the HR patient data acquired from the Infinity Delta monitor as the gold standard is that these monitors are used for continuous patient monitoring at the research site (CHEO); therefore, clinicians depend on the vital sign data displayed by these monitors to routinely assess the patients. Second, we evaluated the SpO_2_ alarm generation performance of the framework as compared with the Masimo SET SmartPod pulse oximeter. Again, this pulse oximeter was selected for comparison because it is routinely used for continuous patient monitoring at the CHEO. RS232 serial ports on both Infinity Delta monitors were connected through Digi International Edgeport4 (Digi International) hardware to a USB port on a computer. Eltima Port Monitor Professional Edition Software v4.x (Eltima Software) was installed on the same computer to read and log data transmitted by each monitor in real time. Thus, a total of 79,200 data points from each physiologic data type were used for analysis. To synchronize data collected from the 2 OEM monitors, these samples were interpolated to obtain one sample every second, resulting in 158,400 data points from each data type. Information regarding patient demographics, inclusion and exclusion criteria, and the detailed methods of data acquisition and data annotation can be found in the author’s earlier research on this data set [44]. A previous study manually counted and categorized patient monitoring alarms [44]. Clinicians, including bedside nurses and neonatologists, validated and categorized the alarms generated by patient monitors. However, manual counting introduces the likelihood of human error. To minimize this likelihood, the process of counting and categorizing the alarms was automated by running the data through a computerized script. This resulted in the identification and categorization of 119 alarms generated by the Masimo pulse oximeter across all 11 patients. These alarms were validated against the clinicians’ original validation and categorization criteria from [44]. The Sn and FAR of the Masimo pulse oximeter were found to be 85% and 46%, respectively.

#### Evaluation Metrics

Data from all 11 patients were used as an input to evaluate each of the four integrated formulations, CDSS #1-4. Leave-one-out cross-validation was used to compute two performance metrics, Sn and FAR. Data from a set of 10 patients were used to tune the components and from the remaining patients to generate alarms. This was repeated 11 times, each time changing the patient for whom the data were left out as a test case.

We then compared the alarm generation performance of each CDSS composition with that of the OEM monitor. Using the OEM monitor’s Sn of 85% and FAR of 46%, we formulated equations (4) and (5) to measure the difference between the Sn and FAR values of the CDSS formulations and the OEM monitor and report that as a percent change. Negative values of percentage change indicate reduction, and positive values indicate increments in Sn and/or FAR. These are reported as (% change in Sn) and (% change in FAR) by equations 4 and 5, respectively.



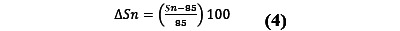





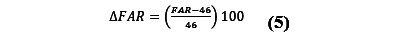



## Results

### Overview

**Figure 5:**
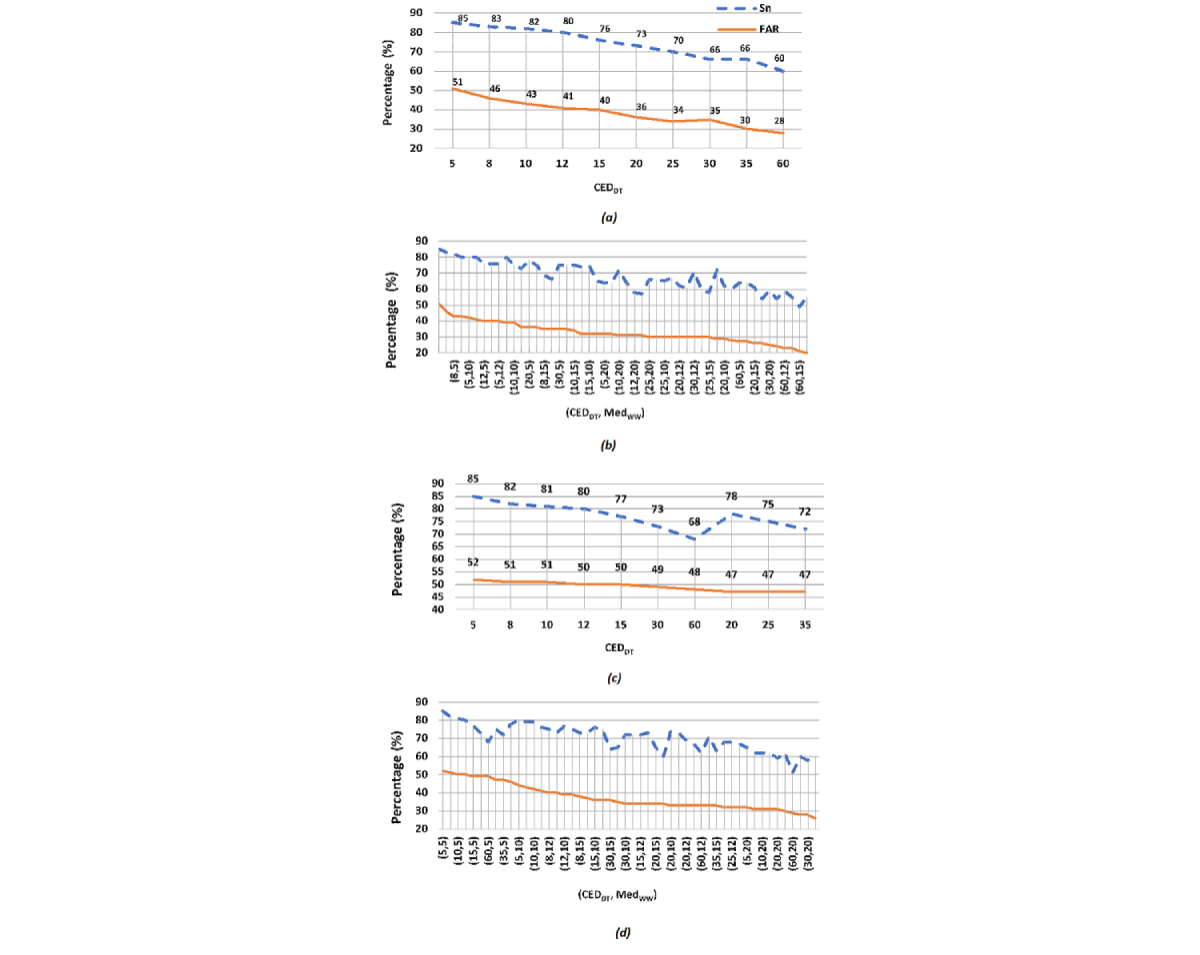
Results of sensitivity (%) and false alarm rate (%) plotted against the relevant tunable parameters CED_DT_ and Med_WW_ for (a) clinical decision support system CDSS #1, (b) clinical decision support system CDSS #2, (c) clinical decision support system CDSS #3 with SQThresh=18, and (d) clinical decision support system CDSS #4 with SQThresh=18. FAR: false alarm rate.

This section presents the performance evaluation results for all four formulations CDSS #1-4 in terms of Sn and FAR, which are averaged across all 11 cross-validation trials. Tables 2 and 3 summarize the pooled results for achieved Sn values of >75% and >80%, respectively. These Sn thresholds were chosen arbitrarily, and other threshold values may be chosen depending on the clinical needs. These tables show the best achievable results expressed as (Sn [% change in Sn], FAR [% change in FAR]) in all four CDSS formulations. The formulations were tabulated based on the inclusion of the AD_MedFilt_ and AD_Diff_ components. Figure 5 shows the graphical results from all four CDSS formulations as linear plots of Sn (%) and corresponding FAR (%) achieved by tuning the parameters Med_WW_, CED_DT_, and *SQThresh*, where applicable to a CDSS. As the configuration parameters of the AD and CED components are varied (tuned), the total number of alarms that are generated also varies. By reporting the performance metrics of Sn and FAR in terms of percentages, we can compare the results across the four CDSS formulations. Here, we compare the best results achieved and tabulated in Tables 2 and 3.

**Table 2:** The best possible (Sn [% change in Sn], FAR ([% change in FAR]) achieved in clinical decision support systems #1-4, where sensitivity≥75%. Tunable parameters are specified for each case.

AD_MedFilt_^a^	AD_Diff_^b^	
Yes	CDSS^c^ #4 Med_WW_^d^=10, CED_DT_^e^=15, SQThresh^f^=18: (76 [−10.5%], 36 [−21.7%])	CDSS #2 Med_WW_=15, CED_DT_=12: (75 [−11.7%], 32 [−30.4%])
No	CDSS #3 CED_DT_=20, SQThreshf=18: (78 [−8.2%], 47 [21.7%])	CDSS #1 CED_DT_=15: (76 [−10.5%], 40 [−15%])

^a^AD_MedFilt_: AD median filter component.

^b^AD_Diff_: AD differential filter component.

^c^CDSS: clinical decision support systems.

^d^Med_WW:_ size of the sliding window of the median filter.

^e^CED_DT_: alarm annunciation delay.

^f^SQThresh: the ordered set of thresholds.

**Table 3:** The best possible (Sn [% change in Sn], FAR [% change in FAR]) achieved in clinical decision support systems #1-4, where sensitivity ≥80%. Tunable parameters are specified for each case.

AD_MedFilt_	AD_Diff_	
	Yes	No
Yes	CDSS^a^ #4 Med_WW_^b^=10, CED_DT_^c^=5, SQThresh^d^=18: (80 [−5.8%], 44 [−4.3%])	CDSS #2 Med_WW_=10, CED_DT_=10: (80 [−5.8%], 39 [−15.2%])
No	CDSS #3 CED_DT_=12, SQThresh=12: (82 [−3.5%], 50 [8.6%])	CDSS #1 CED_DT_=12: (80 [−5.8%], 41 [−10.8%])

^a^CDSS: clinical decision support systems.

^b^Med_WW:_ size of the sliding window of the median filter.

^c^CED_DT_: alarm annunciation delay.

^d^SQThresh: the ordered set of thresholds.

### CDSS #1

The best achievable result for CDSS #1 is (Sn, FAR)=(80, 41) and is obtained when CED_DT_=12, where Sn≥80%. If Sn is only required to be ≥75%, then the best achievable performance becomes (Sn, FAR)=(76, 40) when CED_DT_=15. The FAR (40%) was 15% less than that of the OEM’s FAR (46%). This is achieved at the cost of decreasing Sn (76%) by 10.5% than the Sn of the OEM (85%).

### CDSS #2

Table 2 shows that the best achievable result for CDSS #2 is (Sn, FAR)=(80, 39) when Med_WW_=10 and CED_DT_=10, where Sn≥80%. In this formulation, Sn≥80% was achievable only when Med_WW_≤10. If Sn is allowed to be ≥75%, then the best achievable performance becomes (Sn, FAR)=(75, 32) when Med_WW_=15 and CED_DT_=12.

### CDSS #3

In CDSS #3, with the AD_Diff_ component configured with *SQThresh*=6, the best achievable result for (Sn, FAR)=(86, 52) with CED_DT_=5. The CDSS performance was worse for all other CED_DT_ thresholds at *SQThresh*=6. Although this CDSS performs with an improved Sn (86%) as compared with the OEM’s Sn (85%), the cost is an increase of 13% in the FAR (52%) as compared with the OEM’s FAR (46%).

With the AD_Diff_ component configured with *SQThresh*=12, the best achievable (Sn, FAR) is (82, 50) when CED_DT_=12 for both values of the required Sn≥80% and Sn≥75%. The CDSS performance was worse for all other CED_DT_ thresholds at *SQThresh*=12. When the AD_Diff_ component is configured with *SQThresh*=18, the best achievable result for (Sn, FAR)=(80, 50), with CED_DT_=12 with a threshold of Sn≥80%, and the best achievable result for (Sn, FAR) is (78, 47), with CED_DT_=20 while maintaining Sn≥75%. Thus, CDSS #3 was not able to beat the OEM monitor’s FAR (46%) at any of the parameter settings that were tested.

### CDSS #4

When the AD_Diff_ component of CDSS #4 is configured with *SQThresh*=6 and the sensitivity requirement is ≥80%, the best achievable result for (Sn, FAR)=(84, 52) when Med_WW_=5 and CED_DT_=5. When Sn≥75%, the best achievable (Sn, FAR)=(75, 40) when Med_WW_=10 and CED_DT_=12. Med_WW_>10 resulted in lower (Sn, FAR) values, where Sn<75. If the AD_Diff_ component is configured with *SQThresh*=12 and Sn≥80%, then the best achievable result for (Sn, FAR)=(82, 49) with Med_WW_=5 and CED_DT_=12. When Sn≥75%, the best achievable (Sn, FAR)=(75, 37) is obtained when Med_WW_=12 and CED_DT_=12. Med_WW_>12 resulted in lower (Sn, FAR) values, where Sn<75. Table 3 shows the results from CDSS #4, where the AD_Diff_ component is configured with *SQThresh*=18 and Sn≥80%, and the best achievable result (Sn, FAR)=(80, 44) is obtained when Med_WW_=10 and CED_DT_=5. When Sn≥75%, the best achievable result (Sn, FAR)=(76, 36) is obtained when Med_WW_=10 and CED_DT_=15. Med_WW_>12 resulted in lower (Sn, FAR) values, where Sn<75.

## Discussion

### Principal Findings

The overarching contribution of this study is the illustration of dynamic framework models and their evaluation using clinical data. In this section, we also discuss how this evaluation leads to the validation of the six framework features (*f1*) to (*f6*).

### Framework Evaluation

As described in the *Evaluation* section, the data set used in this evaluation contained 119 alarms across all 11 patients in this study. This data set represents a unique and valuable resource because it includes the detailed annotations of artifacts, alarms, clinical events, clinical interventions, and observations. The patients in our study represented a neonatal population with varying disease severity, weight, and gestational age. Although such a wide range of patients provides for the development of widely applicable rules, as discussed above, many decision thresholds are required to be patient centric. For example, one patient was far more ill than the other 10 patients, with 32% of the associated clinical events. Other limitations of the data set include a possible ambiguity in categorizing alarms as true versus false, especially in cases where the SpO_2_ reading hovers around the OEM monitor’s alarm threshold setting. In this study, such indeterminate alarms were categorized as false alarms. The study sample size was limited because of hospital logistics and resources. In the future, a larger sample size could facilitate subgroup analyses with division based on clinical characteristics, weight, and gestational and chronological age of infants.

From the evaluation results presented in Table 2 under the criterion that Sn≥75%, we infer that CDSS #2 results in the best achievable performance of (Sn, FAR)=(75,32) when Med_WW_=15 and CED_DT_=12. Although a considerable reduction in Sn was observed (11.7%), this parameter combination resulted in a significant reduction in FAR (30.4%). From Table 3, we conclude that CDSS #2 also gives the best possible performance of Sn=80% and FAR=39%, representing percentage reductions of 5.8% and 15.2% for Sn and FAR, respectively. Therefore, CDSS #2 is considered the optimal formulation out of all four CDSS because of the largest reduction in FAR while maintaining Sn≥80%. The optimal parameters for this formulation were Med_WW_=10 and CED_DT_=10.

The results of CDSS #1 illustrate the effects of varying the CED decision threshold (CED_DT_) on the performance of CDSS. By adjusting this threshold, the system could be made more conservative or permissive, leading to an explicit trade-off between Sn and FAR. This CED_DT_ is patient-centered and may be adjusted depending on the severity of disease and clinical resources available, for example, the nurse-to-patient ratio may differ in the NICU versus that in the general ward. A comparison of the results from CDSS #1 and #2 indicates that the use of AD_MedFilt_ significantly improved both the Sn and FAR of the CDSS. As expected, increasing the median filter width reduced both Sn and FAR because the median filter smoothed out transient SpO_2_ values. The range of median filter widths was evaluated in combination with a range of CED_DT_ values seeking the combination that provided the greatest decrease in FAR while maintaining a Sn≥80% or ≥75%. Although these Sn thresholds were somewhat arbitrary, they reflect the need to detect the majority of true clinical events.

CDSS #3 and #4 leveraged data fusion via an AD_Diff_ component to identify periods of low signal quality. Clifford et al [45] recommended that an SQI be generated for each datum when a known error rate is available for calibration. Following this, we hypothesized that by computing the error rate from the combined information from two different sensor modalities, PR from SpO_2_ and HR from ECG, an SQI signal could be generated and increased performance would be achievable. The results for three different AD_Diff_ threshold values failed to demonstrate an improved performance. In fact, the frequency of all three types of alarms, namely, true, missed, and false, increased with the use of AD_Diff_. A close inspection of the generated alarms revealed the fragmentation of previously contiguous alarms into more alarms of shorter duration. This was due to the instantaneous masking of individual SpO_2_ values because of transient disparities between HR and PR, which are not necessarily associated with prolonged periods of low signal quality. We observe that an incremental trend in *SQThresh* values, that is, from 6 to 12 to 18, demonstrates a decreasing trend in Sn and FAR percentages in both CDSS #3 and #4. In future work, the CED algorithm may be modified to process the SQI in a variety of ways that may lead to improved performance. Suggestions for future exploration include either retaining the previous alarm state during periods of low signal quality or appraising cumulative SQI values instead of instantaneous ones.

In summary, dynamic framework modeling showed that the lowest achievable FAR was 39% at a sensitivity of 80%, when compared across all four CDSS formulations.

### Framework Features

The four dynamic CDSS formulations serve to validate the 6 framework features (*f1*) to (*f6*) as follows:

*(f1)* Flexible in serving the differing needs of patient populations from different types of critical care units through generalization and customizability. The CRM includes several fields to generalize and customize each component, for example, Data.Type and Data.SQType. Although the data in this validation study were collected at the NICU, the inherent flexibility of the framework can accommodate various types of data streams acquired from other types of critical care units. Similarly, the component-based nature of this framework allows for the creation of CED components relevant to different clinical domains and for their integration with the most appropriate available AD components. As a result, the components catalog, dynamic framework models, and analyses are not restricted in application to the NICU. This could be demonstrated using future experiments based on data from other units, whether gathered specifically for this research or taken from repositories such as Physio Net [46].

(*f*2) Reusable across multiple types of physiological data harvested by different OEM monitors: The configuration interface of each component permits the setting of OEM-specific and Data.Type-specific values such that the same component may be applied to various physiological data types arising from different OEM monitors. For example, the artSyms configuration parameter allows the AD_DIL_ component to identify artifacts flagged by different OEM monitors. AD components selected from the catalog were used to process different physiological streams acquired by different OEM monitors in various experiments. For example, the AD_DIL_ component is used to process the HR from the Dräger OEM monitor and SpO_2_ and PR from the Masimo OEM pulse oximeter. This validates the reusability of the framework and its components across multiple types of physiological data harvested by different OEM monitors.

(*f3*) Standardized definitions of SQI that promote interoperability between independently developed components: The CRM defines standardized types of SQI, such as, “continuous,” “rank,” and “binary.” These experiments used multiple components to generate the SQI. These components were developed based on the current algorithms identified in the literature review. For example, the AD_Diff_ component is derived from the work of Yu et al [47] and applied to the HR and PR streams in experiments 3 and 4, whereas the CED component leverages the ideas of threshold modification and alarm annunciation delays that were introduced in previous studies [40-43]. These experiments demonstrate the integration of components that were developed independently and whose interoperability is facilitated through the use of standardized SQI, as defined in the framework’s CRM.

(*f4*) Reusability and scalability by cascading, mixing, and matching several AD and CED components in various combinations: By requiring all component interfaces to conform to the standardized CRM, interoperability is promoted, allowing for component reuse and the creation of highly complex pipelines leveraging simple and well-tested components. Each of the four models represented a different component composition. The analyses in each composition vary in scale through the reuse and cascading of components. This mixing and matching are made possible by the adherence of each component to the CRM. Comparing the flowcharts in Figures 3 and 4, there is an increase in the number of instantiations of the AD_DIL_ component from 2 to 4 between CDSS #1 and #3. This demonstrates that the framework supports reusability and scalability by cascading, mixing, and matching several components.

(*f5*) Customizability to evaluate and compare the performance of multiple combinations of independently developed components on offline and potentially real-time patient data when integrated with clinical workflows: A literature review reveals that AD algorithms are typically developed and validated in offline environments [28]. This study illustrates the dynamic framework evaluation using real patient data offline. This validates the use of the framework as a test bed for multiple combinations of independently developed components. Once a combination is affirmed to satisfy clinical needs through offline testing, that combination can then be evaluated in a real-time environment using the middleware technology. In this way, the transition to real-time clinical implementation and validation is facilitated. A number of studies have suggested the introduction of delays in alarm annunciation to reduce FARs. This strategy is expected to reduce the FAR. However, there is a lack of quantitative evaluation in terms of the impact of such a strategy on Sn and FAR. The framework developed here promotes and enables such a quantitative study design, as demonstrated by the experiments developed here. In fact, it was found that such strategies failed to suppress false alarms while maintaining a sufficiently high Sn. This shows that the customizability of the framework allows for performance evaluation and comparison of multiple combinations of independently developed components on offline and potentially real-time patient data when integrated with clinical workflows.

(*f6*) Standardized component interfaces that can potentially support real-time clinical implementation of AD: If independent research and OEM groups choose to implement their algorithms within the context of the framework, that is, adhering to the CRM, then it is more likely that these algorithms will reach clinical implementation because the CRM supports interoperability between all components. Furthermore, the framework simplifies information technology (IT) requirements for hospitals because it provides a unified functional environment in which all AD and CED components required by multiple critical care units can be supported and executed. Finally, the framework facilitates the testing and validation of new algorithms across different clinical settings, populations, critical care units, and pathologies. This will make the system more robust and therefore more likely to be adopted [48]. There is a paucity of CDSS for real-time clinical implementation. One hurdle to their clinical adoption is the requirement to transform complex algorithms for real-time implementation. By implementing the required algorithms within the framework, the algorithms will be made suitable for execution in real time. The four experiments were implicitly designed to run the framework components in a real-time streaming environment. The composition of the analysis in each experiment was evaluated using a simulated real-time environment. As a result, with negligible reformulation, the optimal framework composition resulting from this evaluation can be integrated within clinical workflows. Therefore, we conclude that *the standardized component interface design warranted by the CRM supports real-time clinical implementation of AD within CDSS*.

### Conclusions

This research evaluated a novel AD framework that standardizes the interoperability of AD and CED algorithms for integration within the CDSS. The framework provides a unique test bed with the ability to create and integrate new AD compositions by mixing and matching independently developed or decoupled AD components with CED components that are designed to deliver specific clinical outcomes. This study validates the use of the AD framework in a clinical study using real patient data from the NICU. Several combinations of AD and CED components were evaluated, thereby illustrating the validity of the six framework features, namely, *f1-f6*, including flexibility, reusability, standardization of SQI, scalability, customizability, and support for real-time implementation.

Future work will include the implementation of a wide range of AD and CED components to further leverage the interoperability provided by the CRM. Although the CRM has been developed following an extensive review of the literature that summarizes the requirements, provisions, and configurations for many existing AD algorithms, it is expected that the CRM will continue to evolve as a wide variety of new AD and CED algorithms with differing data needs are implemented as components within this framework. Further validation of the framework can be conducted by independent research groups. The clinical benefits of this research will be broadly realized through the integration of the framework in real-time CDSS to enhance the quality of data analytics. In this way, framework implementation within clinical workflows offers the potential to improve the quality of care for patients and clinicians in critical care.

## Abbreviations

AD: artifact detection
CDSS: clinical decision support systems
CED: clinical event detection
CHEO: Children’s Hospital of Eastern Ontario
CRM: common reference model
DIL: designed to deinterlace
ECG: electrocardiography
FAR: false alarm rate
HR: heart rate
IT: information technology
NaN: Not-a-number
NICU: neonatal intensive care unit
OEM: original equipment manufacturer
PDA: patient data acquisition
PR: pulse rate
SpO2: peripheral oxygen saturation
SQI: signal quality indicator
